# Taurine and glutamine supplementation in aging: systemic mechanisms, exercise interactions, and modulation of muscular and neurobiological pathways

**DOI:** 10.3389/fphys.2026.1809107

**Published:** 2026-05-04

**Authors:** Zhigang Chen, Zhiwei Niu

**Affiliations:** 1 College of Physical Education, Xinjiang Hetian College, Xinjiang, China; 2 Department of Sports, Xinyang Vocational Technical College, Xinyang, China

**Keywords:** aging, exercise, glutamine, protein metabolism, taurine

## Abstract

The senescence of the neuro-skeletal muscle system is typified by a gradual deterioration in muscle mass, strength, neuromuscular efficacy, and cognitive capabilities, which is further exacerbated by metabolic dysregulation and heightened vulnerability to fatigue. The identification of efficacious strategies aimed at reversing or mitigating these deficits is paramount for the promotion of healthy aging. Taurine, glutamine, and physical exercise emerge as promising modulators of cellular homeostasis, exhibiting synergistic potential to address multiple pathways implicated in age-associated decline. This review amalgamates mechanistic insights into the effects of taurine and glutamine supplementation, in conjunction with exercise regimens, on protein metabolism, mitochondrial functionality, muscle fatigue biomarkers, neuroinflammation, redox equilibrium, and body composition throughout the aging process. We examine the role of taurine in preserving calcium homeostasis, enhancing mitochondrial stability, and mitigating oxidative stress; the function of glutamine in sustaining nitrogen balance, regulating immune responses, and facilitating energy metabolism; and the ability of exercise to stimulate critical signaling cascades and antioxidant networks. The integration of taurine and glutamine supplementation with systematically designed exercise regimens may yield a holistic, multi-faceted strategy for counteracting neuro–skeletal muscle aging and augmenting overall functional capacity in the elderly population.

## 1 Introduction

The steady growth of the global older population has intensified scientific attention toward biological processes that erode functional capacity and compromise independence with aging. As life expectancy increases, age-associated declines in muscle strength, metabolic flexibility, immune responsiveness, and cognitive resilience have become major contributors to morbidity and diminished quality of life. These changes are not merely isolated physiological events; rather, they reflect a gradual, systemic loss of adaptability that reduces the capacity of older adults to tolerate physical, metabolic, and environmental stressors (Kirkwood, 2005). Preserving physiological reserve and slowing the trajectory of functional decline have therefore become critical goals in public health. Within the broad spectrum of age-related changes, two closely linked processes create substantial vulnerability in later life, sarcopenia and immunosenescence. Sarcopenia represents the gradual reduction of muscle mass, strength, and neuromuscular efficiency, a decline that progresses silently over time yet exerts marked consequences for mobility, balance, and metabolic health (Lutz and Quinn, 2012). At the same time, immunosenescence reshapes the immune landscape by weakening adaptive responses, exaggerating inflammatory reactivity, and diminishing the ability to mount effective defenses against pathogens or vaccinations (Falahi et al., 2025). Both processes are fueled by chronic low-grade inflammation, oxidative stress, mitochondrial dysfunction, and impaired cellular repair pathways (Liang et al., 2022). These disturbances collectively create an environment in which older adults experience slower recovery, and higher susceptibility to metabolic and functional decline (Hébert, 1997).

Regular physical activity and structured exercise training are among the most effective non-pharmacological strategies to counteract these age-related biological disturbances. Exercise can improve mitochondrial function, enhance metabolic flexibility, reduce chronic low-grade inflammation, and help preserve skeletal muscle mass and strength. Consequently, exercise is widely promoted as a cornerstone intervention to delay or attenuate sarcopenia, immunosenescence, and the functional impairments that accompany aging.

Exercise remains the most powerful non-pharmacological intervention capable of counteracting many of these aging-related trajectories. Aerobic, resistance, and multicomponent training improve muscle function, support metabolic health, and restore aspects of immune regulation across diverse older populations (Pedersen and Saltin, 2015). Despite these benefits, the biological response to exercise is often dampened in aging due to reduced mitochondrial efficiency, lower anabolic sensitivity, impaired antioxidant capacity, and chronic inflammatory signaling. As a result, older adults commonly experience delayed recovery, attenuated gains in muscle strength or aerobic capacity, and limited adaptations compared with younger individuals. Despite these well-documented benefits, exercise alone may be insufficient to fully reverse or prevent the complex biology of aging. Older adults often display reduced adaptive capacity, anabolic resistance, impaired nutrient utilization, and persistent inflammatory and oxidative stress burdens that can limit the extent of exercise-induced improvements. In addition, comorbidities, frailty, and practical barriers such as pain, fatigue, or limited mobility may restrict the intensity and volume of exercise that can be safely performed. As a result, many individuals do not achieve complete restoration of muscle function, immune competence, or metabolic health through exercise interventions alone.

This “blunted responsiveness” has generated interest in adjunctive nutritional strategies that might enhance or stabilize the physiological environment in which exercise adaptations occur (Pedersen and Saltin, 2015; Clifford, 2019). Within this context, amino acids with regulatory functions not only those involved in protein synthesis have gained increasing scientific attention. Several amino acids act as metabolic substrates, antioxidants, osmolytes, neurotransmitter modulators, or immune-regulating molecules. Unlike macronutrient-based protein supplements, these amino acids influence signaling pathways central to redox control, metabolic homeostasis, mitochondrial stability, and inflammatory balance (Coleman et al., 2020; Wu, 2020). With aging, circulating levels of several regulatory amino acids decline due to alterations in diet, absorption, hepatic metabolism, and reductions in skeletal muscle, the major reservoir of many amino acids. This has raised the possibility that targeted supplementation could partially restore biochemical pathways that weaken with age (Ten Have et al., 2007; Young et al., 2018). Among these compounds, taurine and glutamine have emerged as two biologically compelling candidates. Taurine, one of the most abundant free amino sulfonic acids in excitable and metabolically active tissues, plays essential roles in calcium handling, mitochondrial protection, membrane stabilization, bile acid conjugation, osmoregulation, and modulation of inflammatory and oxidative processes (Schaffer et al., 2010).

Conversely, glutamine, the most prevalent free amino acid in human plasma and skeletal muscle, is integral to nitrogen shuttling, immune cell metabolism, glutathione synthesis, acid–base balance, and intestinal health (Newsholme et al., 2003). Importantly, levels of both taurine and glutamine tend to decline with advancing age, at the same time that the physiological systems they support, redox regulation, immune competence, muscle integrity, and metabolic flexibility, become increasingly strained (Hädel et al., 2013; Singh et al., 2023). Despite their biological relevance, human evidence examining taurine and glutamine specifically within the context of exercise and aging remains fragmented. Existing clinical trials vary widely in population characteristics, dosing regimens, supplementation duration, and outcome domains. Studies have explored effects in healthy older adults, individuals with metabolic disorders, postmenopausal women, cardiac patients, persons with sarcopenic obesity, and older adults undergoing rehabilitation (Ko et al., 2020; Maleki et al., 2020; Rondanelli et al., 2021; de Nóbrega et al., 2024; Nie et al., 2025). Among these potential adjuncts, the amino acid-related compounds taurine and glutamine have received increasing attention. Both are abundant in human tissues and participate in essential processes such as mitochondrial bioenergetics, osmoregulation, antioxidant defense, immune regulation, and maintenance of muscle and metabolic homeostasis. Emerging evidence suggests that taurine and glutamine may modify several hallmarks of aging and influence the adaptive response to exercise in older individuals. In this review, we summarize current knowledge on the roles of taurine and glutamine in aging biology and exercise. We first outline key mechanisms underlying age-related declines in muscle and immune function, then examine how taurine and glutamine may modulate these pathways and act as adjunct therapies to enhance exercise-induced benefits in older adults.

Moreover, although both compounds have been independently studied, no comprehensive review has systematically compared their roles across functional, metabolic, immunological, and exercise-related domains in aging. This gap provides the central rationale and novelty of the present review. By integrating evidence from randomized and controlled trials on taurine and glutamine supplementation in older adults, and by explicitly examining how these compounds interact with different forms of exercise, this review aims to clarify their potential as complementary strategies to support healthy aging. The review evaluates effects across four major physiological domains such as neuroimmune stability, physical performance, metabolic regulation, and immune responsiveness, while also identifying limitations, safety considerations, and priorities for future research. In doing so, the review establishes a foundation for evidence-informed clinical and practical recommendations and prepares the conceptual groundwork for the mechanistic and trial-based discussions that follow.

## 2 Exercise physiology in aging: foundations for amino acid–enhanced adaptation

Aging gradually disrupts metabolic, inflammatory, and neuromuscular pathways, reducing physiological resilience and raising disease risk. These shifts are reflected in circulating markers such as amino acid levels, cytokines, and redox indicators (Martínez de Toda et al., 2020). Recognizing these patterns helps explain why taurine and glutamine are increasingly examined as nutritional targets in aging research. Across population studies, one of the most consistent biochemical patterns is a gradual reduction in circulating taurine and glutamine concentrations.

Taurine levels decline from midlife onward, likely due to reduced endogenous synthesis, alterations in sulfur amino acid metabolism, and age-related changes in dietary habits (Fernandez et al., 2025). Glutamine availability also falls with age, particularly as skeletal muscle atrophies and immune activation increases metabolic demand. Frail and hospitalized older adults frequently demonstrate low plasma glutamine, reflecting catabolic stress and impaired protein turnover (Wilmore, 2001; Meynial-Denis, 2016). These shifts are clinically relevant because both amino acids play key roles in antioxidant defense, mitochondrial integrity, cellular osmotic regulation, and immune homeostasis, adsystems that progressively lose efficiency with advancing age.

Parallel to these amino acid declines, chronic low-grade inflammation becomes increasingly prominent. Circulating Interleukin-6 (IL-6), Tumor Necrosis Factor-alpha (TNF-α), and C-Reactive Protein (CRP) rise steadily across older adulthood, forming the well-described “inflammaging” phenotype associated with sarcopenia, cardiovascular risk, reduced mobility, and diminished vaccine responsiveness (Franceschi et al., 2018). Higher IL-6 predicts slower gait speed and weaker grip strength, while elevated TNF-α is linked to accelerated muscle protein breakdown and fatigue (Degens, 2010). Given that taurine derivatives can attenuate Nuclear Factor kappa-light-chain-enhancer of activated B cells (NF-κB) signaling and glutamine supports lymphocyte proliferation and cytokine regulation, declining levels of these nutrients may amplify inflammatory drift (Rohde et al., 1996; Sun et al., 2012).

Oxidative stress indices show similar age-related deterioration. Declines in the reduced glutathione/oxidized glutathione ratio indicate compromised redox buffering capacity and heightened vulnerability to mitochondrial injury (Jones, 2008). Increased concentrations of lipid peroxidation markers, such as maldialdehyde (MDA), and DNA oxidation products, including 8-Hydroxy-2’-deoxyguanosine (8-OHdG), correlate with frailty severity, impaired physical function, and greater disability risk (Ruangsuriya, 2021). Taurine helps stabilize mitochondrial electron transport and mitigate Reactive Oxygen Species (ROS) formation, while glutamine serves as a precursor for glutathione synthesis and supports antioxidant enzyme activity; therefore, reduced availability of either compound can intensify oxidative imbalance in aging tissues (Shimada et al., 2015; Aldarini et al., 2017).

**Figure 1:**
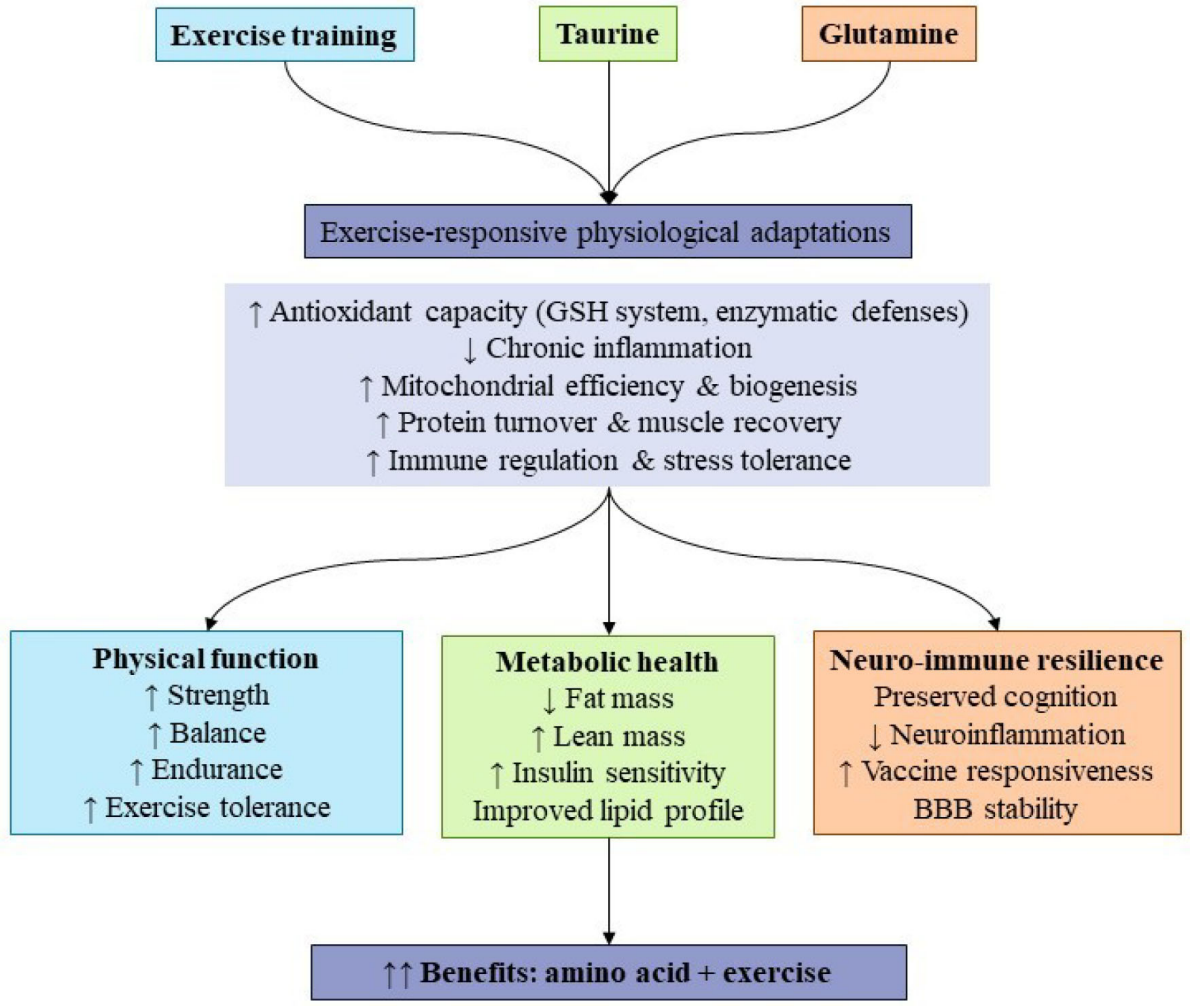
Synergistic effects of exercise with taurine and glutamine on functional outcomes in aging. Exercise training is the primary stimulus for physiological adaptation in older adults, influencing antioxidant capacity, inflammation, mitochondrial efficiency, protein turnover, and immune regulation. Taurine and glutamine supplementation act as supportive modulators that enhance these exercise-responsive pathways, leading to improvements in physical performance, metabolic health, body composition, and neuro-immune resilience. The greatest benefits are observed when supplementation is combined with structured exercise rather than used in isolation.

Neuromuscular biomarkers also undergo predictable declines. Circulating insulin-like growth factor 1(IGF-1) progressively decreases with age and is strongly associated with reduced muscle protein synthesis, mitochondrial biogenesis, and functional capacity (Barclay et al., 2019). Conversely, myostatin levels increase, inhibiting muscle growth and contributing to sarcopenia. Age-associated reductions in mitochondrial enzyme activities, such as citrate synthase and electron transport chain complexes,further impair aerobic capacity and slow recovery after exertion (Ferri et al., 2020) (**Figure 1**).

Within this pro-inflammatory background, exercise represents a uniquely potent countermeasure that can recalibrate immune activity, restore redox balance, and stimulate mitochondrial renewal (Franceschi and Campisi, 2014). Within this landscape, structured exercise remains one of the few interventions with robust, cross-system efficacy. Aerobic, resistance, and multicomponent exercise programs are consistently shown to attenuate inflammatory tone, enhance antioxidant capacity, and improve mitochondrial efficiency, effects that collectively contribute to healthier aging trajectories (Pedersen and Febbraio, 2012; Hood et al., 2019). Because physical activity influences many of the same pathways affected by aging, it provides a biologically logical foundation upon which nutritional strategies may act synergistically.

The anti-inflammatory effects of exercise are particularly relevant to aging physiology. Contractions of active muscle fibers stimulate the release of myokines such as IL-6 (in its anti-inflammatory form), IL-10, and irisin, which collectively suppress chronic inflammation and improve metabolic signaling (Brandt and Pedersen, 2010). These myokine-mediated responses help counter the persistent inflammatory environment characteristic of older adults. Exercise also enhances immune surveillance, improves T-cell function, and modulates innate immune pathways, making it a valuable non-pharmacological option for mitigating immunosenescence (Kohut and SenChina, 2004). Such improvements are especially important because older adults often exhibit impaired vaccine responses, reduced naïve T-cell pools, and a heightened risk of chronic inflammatory disease (Pinti et al., 2016).

Parallel to these immunological effects, exercise exerts powerful benefits on mitochondrial dynamics. Regular physical activity stimulates mitochondrial biogenesis, improves oxidative phosphorylation capacity, and enhances mitophagy, thereby reducing the accumulation of dysfunctional mitochondria that contribute to oxidative stress (Hood et al., 2019). Enhanced mitochondrial efficiency is closely tied to improvements in aerobic performance, metabolic flexibility, and muscle endurance—domains that typically decline with advancing age. Resistance training adds another dimension by increasing muscle cross-sectional area, improving neuromuscular recruitment, and enhancing excitation–contraction coupling, all of which support functional independence in later life (James et al., 2021; Memme et al., 2021).

Given this broad physiological influence, it is not surprising that amino acids capable of modulating redox balance, immune signaling, or metabolic pathways may interact meaningfully with exercise. Nutrients such as taurine and glutamine influence antioxidant buffering, mitochondrial stability, calcium handling, immune cell metabolism, and nitrogen transport (de Oliveira et al., 2016; Jong et al., 2021). Since aging weakens many metabolic and cellular pathways, targeted supplementation may help strengthen exercise-related adaptations by supplying additional substrates or regulatory support during periods of higher physiological demand. This combined approach is supported by the fact that both exercise and these amino acids influence redox balance, inflammatory signaling, mitochondrial function, and muscle metabolism, even though they operate through partly different mechanisms. The potential for synergy arises because exercise creates an adaptive environment in which nutrients are more effectively utilized (Li et al., 2025). For example, training increases amino acid transport into muscle, enhances the activity of antioxidant enzyme systems, and stimulates pathways involved in protein turnover and mitochondrial remodeling (Damanti et al., 2019). This means that supplemental amino acids may exert stronger effects when metabolic signaling is already activated by exercise. Conversely, supplementation alone without a training stimulus may be insufficient to drive functional or metabolic improvements, particularly in older adults with blunted anabolic or immune responses. This conceptual interaction provides the scientific basis for reviewing taurine and glutamine in the context of exercise rather than as isolated interventions.

Beyond metabolic and neuromuscular considerations, exercise also influences cognitive and neuroimmune health. Regular physical activity increases neurotrophic factors such as brain-derived neurotrophic factor (BDNF), enhances cerebrovascular function, and promotes synaptic plasticity through activity-dependent mechanisms (Cotman and Berchtold, 2002). These adaptations occur alongside improvements in blood–brain barrier (BBB) integrity and reductions in neuroinflammation—pathways also implicated in age-related cognitive vulnerability (Laitman and John, 2015). Amino acids with neuromodulatory or antioxidant properties may therefore complement exercise-driven neural benefits by stabilizing redox homeostasis, supporting neurotransmitter metabolism, or modulating inflammatory mediators within the central nervous system (Rajagopal et al., 2016).

Overall, exercise influences a wide range of systems by reducing inflammation, enhancing mitochondrial efficiency, improving metabolic regulation, strengthening immune function, and supporting cognitive health. Taurine and glutamine, through their antioxidant and immunometabolic actions, interact with many of these same pathways. Considering their overlapping biological effects, exploring how these amino acids work alongside exercise offers a mechanistic basis for understanding their potential to enhance physiological adaptation in older adults.

## 3 Taurine supplementation and exercise in older adults: clinical evidence across cognitive, physical, and metabolic domains

Taurine is a sulfur-based amino acid involved in osmoregulation, mitochondrial function, calcium control, and antioxidant protection. Although the body can synthesize it, taurine levels decline with age, contributing to weaker metabolic resilience, heightened inflammation, and reduced neuromuscular performance (Kriebs, 2023). These shifts may underlie poorer physical capacity and slower recovery in older adults. Exercise helps counteract many of these age-related changes by improving redox balance, metabolism, muscle strength, and cognitive health, but it also increases nutrient demands (Lanza et al., 2008).

**Figure 2:**
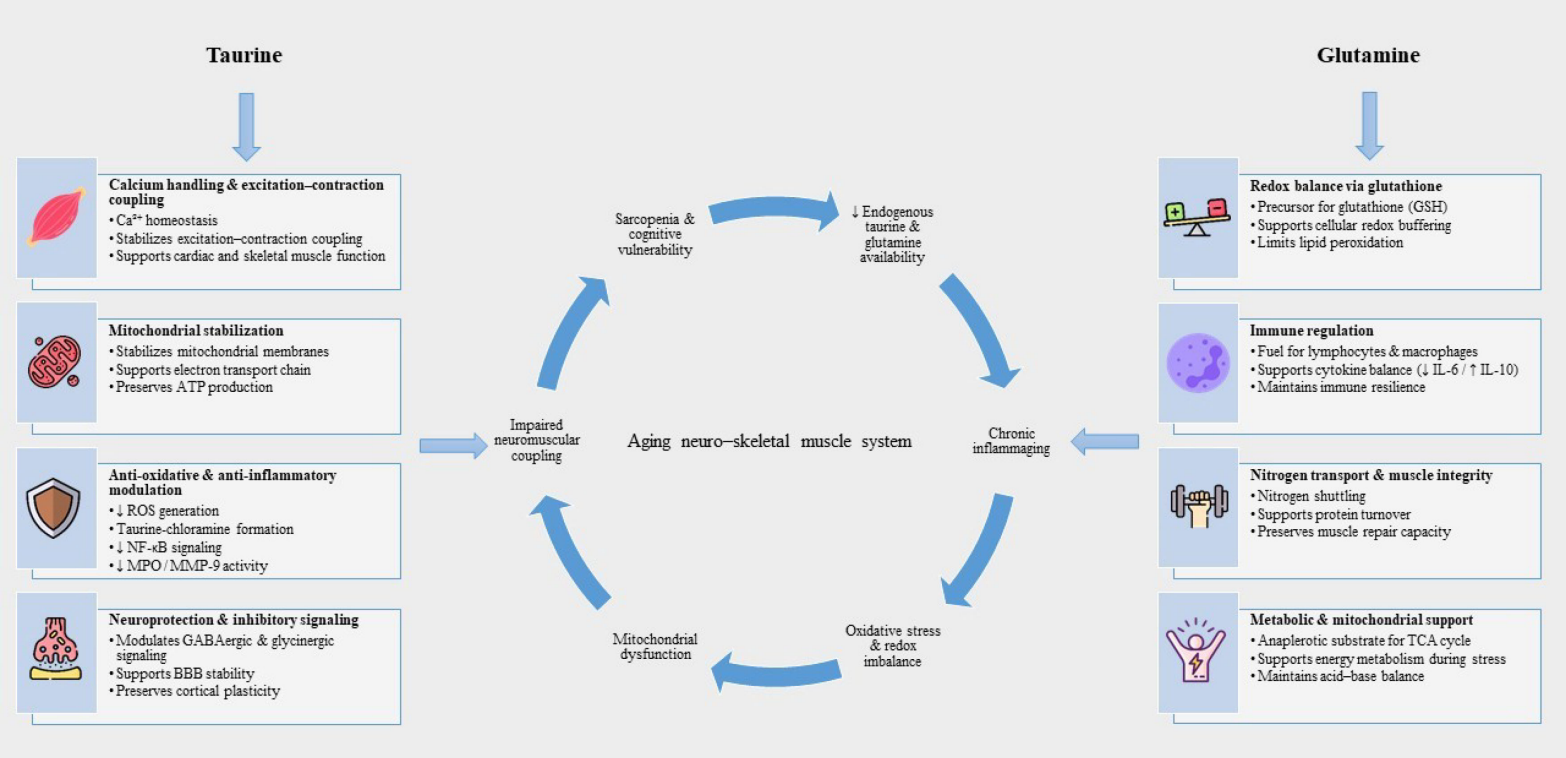
Mechanistic basis of taurine and glutamine actions in neuro-skeletal muscle aging. Aging is accompanied by reduced availability of taurine and glutamine, chronic low-grade inflammation, oxidative stress, mitochondrial dysfunction, and impaired neuromuscular integrity. Taurine supports calcium homeostasis, mitochondrial stability, anti-oxidative and anti-inflammatory signaling, and neuroprotection, whereas glutamine contributes to redox balance through the glutathione system, immune regulation, nitrogen transport, and muscle maintenance. Together, these complementary pathways target key biological processes that deteriorate with advancing age. This image has been designed using resources from Flaticon.com.

Although taurine and glutamine differ in structure and biological roles, both act on key pathways that progressively weaken with aging. Each contributes to antioxidant defense through its own mechanisms. Taurine stabilizes mitochondrial electron flow and generates taurine-chloramine, a compound that helps restrain excessive oxidative activity (Cunningham et al., 1998). Similar to glutamine, Taurine affects inflammatory dynamics. Taurine-chloramine dampens overly aggressive immune activation, while glutamine promotes a more regulated immune environment by sustaining lymphocyte function and supporting anti-inflammatory cytokine profiles such as higher interleukin-10 (IL-10) relative to pro-inflammatory mediators (Schuller-Levis and Park, 2004; Sartori et al., 2018). Mitochondrial maintenance represents another shared node. Taurine assists mitochondrial tRNA modifications, stabilizes respiratory chain activity, and contributes to ATP synthesis under stress (Suzuki et al., 2002). These influences on energy metabolism, oxidative buffering, and immune competence are particularly relevant for older adults, who face declining mitochondrial function and reduced physiological adaptability. In neuromuscular and metabolic pathways, both compounds again play complementary roles. Taurine contributes to calcium-dependent contraction and cardiac electrical stability, supporting efficient muscle performance and cardiovascular responsiveness (Ito et al., 2014). Together, these biological effects help mitigate hallmarks of aging such as sarcopenia, metabolic inflexibility, inflammatory vulnerability, and impaired exercise tolerance (**Figure 2**).

Because taurine participates in these protective pathways and decreases with age, it is increasingly considered a supportive adjunct to exercise in older adults. Recent clinical trials have begun to examine whether taurine supplementation alone or combined with structured exercise can modulate inflammation, support neuromuscular function, and improve physical or cognitive outcomes in aging populations. The characteristics and key findings of these studies are summarized in **Table 1**, which forms the foundation for the following sections.

**Table 1:** Summary of clinical and experimental evidence on taurine supplementation across aging, metabolic, and functional outcomes.

Study design & population characteristics	Sample size & taurine intervention	Main findings	Mechanistic notes	Reference
RCT in elderly women (~83 y) evaluating taurine, exercise, and combined effects over 14 weeks.	n=48; Taurine 1.5 g/day; 4-group design.	Taurine ↓MPO & MMP-9; ETTS improved fitness; cognition declined only in control.	Anti-inflammatory action; synergy with exercise; no BDNF effect.	(Chupel et al., 2021)
Double-blind crossover trial in healthy elderly men (60–69 y) testing acute taurine doses.	n=15; Single-dose 1 g vs 6 g vs placebo.	6 g improved balance, endurance, aerobic capacity & MMSE; 1 g ineffective.	Dose-dependent effects on neuromuscular & cognitive performance.	(Nasimi and Tadibi, 2025)
RCT in elderly women (~83 y) testing taurine + multimodal exercise for 14 weeks.	n=48; Taurine 1.5 g/day; 4-group design.	Exercise ↓TNF-α, IL-6; taurine ↓IL-1β/IL-1ra; CET+TAU improved MMSE.	Reduced inflammation; maintained BBB integrity; cognitive synergy.	(Chupel et al., 2018)
Longitudinal cohort of Japanese adults aged 40–79 year**s** (8-year follow-up)	n=1,454; dietary taurine intake (no supplementation).	Higher taurine intake preserved knee extensor strength; no effect on other fitness tests.	Long-term dietary taurine supports muscle strength maintenance.	(Domoto et al., 2024)
RCT in heart failure patients (60 y, NYHA II–III) pre/post incremental exercise.	Taurine 500 mg ×3/day for 2 weeks.	↓CRP, ↓platelets, ↓CRI-I/II & AC; placebo worsened.	Anti-inflammatory & anti-atherogenic effects around exercise.	(Ahmadian et al., 2017b)
RCT in heart failure patients (60 y, NYHA II–III) pre/post incremental exercise	n=16; 500 mg taurine ×3/day for 2 weeks.	↑ exercise capacity, METs, distance; ↓ QT and ↑ PR interval; improved cardiac electrical function.	Membrane stabilization; improved myocardial efficiency.	(Ahmadian et al., 2017a)
RCT in postmenopausal women (50–79 y).	n=43; 1.5 g/day; 4 groups including exercise + taurine	TE group had greatest fat loss, lean mass gain, LDL & TC reduction.	Exercise–taurine synergy in lipid control	(Buonani et al., 2019)
RCT in women with T2D (~53 y).	n=40; taurine 3 g/day; TRX + taurine vs control groups	TT group showed strongest improvements in HbA1c, HOMA-IR, TG, TC & HDL	Enhanced insulin sensitivity with exercise + taurine	(Samadpour Masouleh et al., 2021)
RCT in older women (≥60 y) with sarcopenic obesity	n=35; taurine 3 g/day; 16-week multicomponent training	Taurine + exercise markedly reduced adipocyte area; ↑ REE in exercise groups	Improved adipose remodeling & metabolic activation	(Ortiz et al., 2025)
RCT in older women (60–75 y).	n=35; taurine 3 g/day; 16-week protocol	Exercise (± taurine) improved glucose, insulin & insulin sensitivity; taurine alone minimal	Exercise-driven glucose utilization; reduced circulating monosaccharides	(Abud et al., 2025)
Double-blind crossover trial in active top-aged adults (52–56 y).	n=31; multi−nutrient including taurine for 28 days	↓ IL−6 in both sexes; men: ↓ CK, ↓ pain, ↑ strength; women: ↓ anxiety, ↑ balance	Anti-inflammatory and neuromuscular recovery effects	(Dunn-Lewis et al., 2011)
Animal study in young (3 mo) and aged (12–24 mo) mice	n=60; taurine supplementation compared with environmental enrichment	Taurine restored learning−induced plasticity in aged cortex; maintained SOM+ interneuron response	Improved cortical plasticity independent of GABA/glutamate normalization	(Gawryluk et al., 2024)
Single-blind RCT in HF patients (~60 y).	n=29; 500 mg taurine ×3/day for 2 weeks	↑ exercise time, METS & distance; no changes in placebo	Enhanced myocardial oxygen usage; improved endurance	(Beyranvand et al., 2011)

RCT, Randomized Controlled Trial; MPO, Myeloperoxidase; MMP-9, Matrix Metalloproteinase-9; ETTS, Exercise Training + Taurine Supplementation; BDNF, Brain-Derived Neurotrophic Factor; MMSE, Mini-Mental State Examination; TNF-α, Tumor Necrosis Factor-alpha; IL-6, Interleukin-6; CET+TAU, Combined Exercise Training + Taurine; BBB, Blood-Brain Barrier; CRP, C-Reactive Protein; CRI-I, Castelli’s Risk Index-I; CRI-II, Castelli’s Risk Index-II; AC, Atherogenic Coefficient; NYHA, New York Heart Association Classification; METS, Metabolic Equivalents; QT, QT Interval; PR, PR Interval; LDL, Low-Density Lipoprotein; TC, Total Cholesterol; HbA1c, Hemoglobin A1c; HOMA-IR, Homeostatic Model Assessment of Insulin Resistance; TG, Triglycerides; HDL, High-Density Lipoprotein; REE, Resting Energy Expenditure; GABA, Gamma-Aminobutyric Acid; HF, Heart Failure. ↓ Decrease, ↑ Increase.

### 3.1 Pharmacokinetics

Taurine and glutamine exhibit distinct yet complementary pharmacokinetic and tissue-distribution profiles that shape their physiological actions in older adults. These characteristics, absorption, transport, metabolic handling, and clearance, directly influence how each amino acid interacts with exercise, inflammation, redox balance, and neuromuscular aging. Understanding these processes provides a mechanistic bridge between basic biochemistry and the performance and health outcomes observed in later life. Taurine is absorbed primarily in the small intestine through sodium-dependent active transport systems, after which it circulates largely in its free form and displays a strong tendency to accumulate within excitable tissues such as cardiac and skeletal muscle, retina, and the central nervous system (Lambert et al., 2015). Intracellular uptake relies heavily on the taurine transporter (TauT), a high-affinity, low-capacity system expressed widely but particularly concentrated in heart, muscle, and neural tissue (De Luca et al., 2015). Age-related reductions in TauT density and altered cellular ion gradients can decrease taurine uptake efficiency, leading to lower tissue concentrations despite stable plasma levels (Fernandez et al., 2025).

Exercise interacts with these pharmacokinetic features in meaningful ways. Acute physical activity increases skeletal muscle blood flow and transporter trafficking, potentially enhancing taurine uptake and retention, whereas chronic training appears to upregulate TauT expression and improve intracellular buffering capacity (Rutherford et al., 2010). As exercise modifies the kinetics of both taurine and glutamine, a closer look at how these amino acids are absorbed and retained helps clarify their distinct physiological roles. Taurine loading occurs slowly because of the saturable nature of TauT, producing incremental tissue accumulation over days to weeks (Pasantes-Morales, 2017). In contrast, glutamine peaks rapidly but returns to baseline quickly due to high metabolic turnover (Matés et al., 2009).

This divergent kinetics may explain why taurine tends to influence endurance, neuromotor, and cardiometabolic domains, whereas glutamine more consistently affects immune regulation and glutathione-related redox pathways. Together, these pharmacokinetic patterns clarify why taurine and glutamine produce domain-specific benefits in aging: taurine through sustained tissue retention and neuromuscular stabilization, glutamine through rapid metabolic support of immune and antioxidant systems. Such distinctions provide a mechanistic foundation for interpreting trial outcomes and underscore the importance of tailoring supplementation strategies to physiological context in older adults.

### 3.2 Taurine and neuroimmune aging: effects on cognition, inflammation, and BBB integrity

Aging disrupts antioxidant balance, mitochondrial function, immune regulation, and metabolic flexibility, reducing resilience and slowing recovery (Promislow et al., 2022). Within this altered physiology, taurine and glutamine are notable because they influence pathways that decline with age, including redox control, inflammation, and neuromuscular and immune support, making them relevant to exercise-related adaptations in older adults (Walsh et al., 1998; Merckx and De Paepe, 2022). Taurine, although not incorporated into proteins, is abundant in excitable tissues such as the brain, skeletal muscle, and myocardium. Its multifunctional properties include the regulation of cellular osmolarity, modulation of calcium handling, support of mitochondrial activity, facilitation of bile acid conjugation, and participation in antioxidative pathways that protect cells from excessive reactive species (Huxtable, 1992).

Taurine also influences inhibitory neurotransmission via Gamma-Aminobutyric Acid (GABA) and glycine systems, contributing to neural stability under metabolic or oxidative stress (Oja and Saransaari, 1996). Evidence suggests that endogenous taurine availability progressively declines with advancing age, partly due to reduced synthesis and lifestyle-related dietary variations (Dawson et al., 1996). Lower intracellular and plasma levels have been associated with impaired mitochondrial function, less efficient excitation–contraction coupling, and greater susceptibility to oxidative and inflammatory stressors in older individuals (Marcinkiewicz and Kontny, 2014).

Because exercise challenges many of the same systems—mitochondrial adenosine triphosphate (ATP) generation, redox homeostasis, calcium flux, and contractile efficiency—the biological overlap between taurine-dependent pathways and exercise-induced stress creates a logical basis for exploring how taurine might enhance the adaptive benefits of training in older adults. Glutamine plays an equally central, but mechanistically distinct, role in aging biology. As the most abundant free amino acid in circulation, it serves as a major metabolic substrate for rapidly proliferating immune cells, sustains intestinal barrier integrity, assists in nitrogen transport, and contributes to acid–base homeostasis (Newsholme, 2001).

Age-related dysregulation of the neuroimmune system, characterized by chronic low-grade inflammation, shifts in redox status, and diminished neuronal resilience, plays a central role in the gradual decline of cognitive function in later life (Gu et al., 2025). These systemic alterations frequently coincide with structural vulnerabilities, including reduced stability of the BBB and attenuated synaptic plasticity (Erdő et al., 2017). Given its antioxidant, osmotic, and neuromodulatory properties, taurine has attracted attention as a nutritional compound that may counteract several of these biological disturbances (Pasantes-Morales and Schousboe, 1997). In the following paragraphs, evidence from human trials and experimental models is reviewed to clarify how taurine may influence cognition, neuroinflammation, and mechanisms linked with BBB function during aging.

Chupel et al (Chupel et al., 2021). evaluated whether taurine supplementation, either alone or in combination with exercise, could modulate inflammatory and neurodegenerative biomarkers in very old women. Their 14-week randomized design included four conditions: exercise training, taurine ingestion (1.5 g/day), combined taurine plus exercise, and a non-intervention control. Measurements encompassed oxidative enzymes such as myeloperoxidase (MPO), indicators of extracellular matrix degradation (MMP-9), neurotrophins BDNF and Nerve Growth Factor (NGF), circulating immune cell subsets, and cognitive status assessed by the Montreal Cognitive Assessment (MoCA). Taurine alone produced meaningful reductions in MPO and MMP-9, suggesting a lowering of inflammatory and proteolytic activity commonly elevated in advanced aging. Although neurotrophin concentrations remained largely unchanged, taurine prevented the cognitive decline observed in the control group (Chupel et al., 2021). The most pronounced gains in both cognitive and physical outcomes appeared when taurine was paired with exercise, indicating a potential synergistic effect. Minor adjustments in lymphocyte and monocyte counts also pointed toward more favorable immune balance. Altogether, these findings suggest that taurine may help counter inflammatory progression and preserve cognitive performance in elderly women, particularly when used alongside regular physical activity (Chupel et al., 2021).

In a complementary line of inquiry, Nasimi et al (Nasimi and Tadibi, 2025). assessed whether acute taurine ingestion could influence motor and cognitive function in older men. Using a double-blind crossover approach, each participant completed three separate sessions, receiving 1 g taurine, 6 g taurine, or placebo one hour before testing. Cognitive status (MMSE), balance (TUG), muscular endurance, and cardiorespiratory capacity (6-minute walk test) were examined in each condition. A clear dose-dependent pattern emerged: only the 6 g dose yielded consistent improvements across all outcomes. High-dose taurine resulted in markedly better MMSE scores than placebo and low-dose conditions, accompanied by enhancements in balance, endurance, and walking distance. These changes imply improved neuromotor coordination and metabolic efficiency (Nasimi and Tadibi, 2025). The absence of measurable effects with the 1 g dose suggests the presence of a physiological threshold required for acute taurine responsiveness. Although mechanistic pathways were not directly explored, the rapid improvements align with taurine’s proposed effects on mitochondrial function, calcium handling, and antioxidative support. Accordingly, high-dose taurine may offer a short-term means of elevating cognitive–motor capacity in older men, though its long-term efficacy warrants further examination (Nasimi and Tadibi, 2025).

In an earlier study, Chupel et al (Chupel et al., 2018). also investigated the interplay between taurine, exercise, systemic inflammation, and peripheral markers associated with BBB function in elderly women. Following the same four-arm structure—exercise, taurine alone, combined taurine plus exercise, and control—participants completed 14 weeks of intervention. The authors assessed a range of serum cytokines (IL-1β, IL-6, IL-10, TNF-α), the IL-1β/IL-1 ratio, and two biomarkers linked with BBB permeability: S100β and neuron-specific enolase (NSE) (Chupel et al., 2018). Exercise, whether alone or combined with taurine, generated the strongest anti-inflammatory response, including reductions in IL-6, TNF-α, and several pro- to anti-inflammatory cytokine ratios. Taurine by itself decreased the IL-1β/IL-1 ratio, indicating selective immunomodulation. S100β concentrations remained stable in all intervention groups, whereas a slight increase emerged in controls, implying preserved BBB integrity with taurine or exercise. NSE levels increased only among participants receiving taurine alone—a finding interpreted cautiously as a potential metabolic adjustment rather than neuronal distress. Importantly, cognitive performance (MMSE) improved exclusively in the combined taurine + exercise condition. Together, these data highlight coordinated benefits across neuroimmune and BBB-related pathways, again suggesting that taurine’s most robust effects arise when integrated with structured physical activity (Chupel et al., 2018).

Further insight into taurine’s role in neural plasticity derives from an experimental study by Gawryluk et al (Gawryluk et al., 2024). Using a mouse model of barrel cortex learning, the authors explored how aging affects experience-dependent plasticity and whether taurine supplementation or environmental enrichment could mitigate these deficits. Older mice exhibited markedly reduced cortical taurine levels and showed limited capacity to induce plastic changes following tactile conditioning. Both taurine supplementation and enriched housing restored the animals’ ability to expand the functional representation of the trained whisker row, reaching levels comparable to young controls (Gawryluk et al., 2024). Notably, taurine’s beneficial effects did not depend on correcting the age-related imbalance in glutamate/GABA signaling. Instead, taurine increased activation of somatostatin-expressing interneurons, a hallmark of effective plasticity in young mice. These findings point to a mechanism through which taurine enhances inhibitory microcircuits essential for learning-related remodeling. The clear age-graded decline in endogenous taurine further supports the rationale for nutritional supplementation as a means to maintain neural adaptability (Gawryluk et al., 2024). Overall, this experimental work reinforces the possibility that taurine can help counteract the rigidity of aging neural circuits.

Taken together, human and animal investigations converge on the notion that taurine supports neuroimmune stability during aging. Supplementation reduces pro-inflammatory mediators, limits tissue-degrading enzymatic activity, and appears to preserve BBB-related markers. Cognitive improvements are modest with taurine alone but become more evident when accompanied by exercise. Experimental findings add another dimension, demonstrating that taurine can restore experience-dependent cortical plasticity through mechanisms involving inhibitory interneuron engagement. Collectively, these observations suggest that taurine influences both systemic and neuronal processes tied to age-related cognitive vulnerability, positioning it as a potentially valuable adjunct within lifestyle-based strategies aimed at promoting healthy brain aging.

### 3.3 Taurine and physical performance: effects on strength, balance, endurance, and exercise capacity in aging

Age-associated reductions in muscle strength, balance, mobility, and overall exercise tolerance substantially contribute to functional decline and loss of independence in later life (Lauretani et al., 2003). As skeletal muscle becomes increasingly susceptible to oxidative stress, mitochondrial inefficiency, and impaired excitation–contraction coupling, even basic daily tasks require greater physiological effort (Grevendonk et al., 2021). This heightened vulnerability has intensified interest in nutritional compounds capable of supporting muscle metabolism and cardiopulmonary function.

Taurine, through its regulatory actions on calcium handling, anti-inflammatory pathways, mitochondrial integrity, and cardiac physiology, has gained attention as a potential nutritional strategy for preserving physical performance with aging (Thirupathi et al., 2020). The following section synthesizes evidence from longitudinal and clinical trials evaluating how dietary taurine or short-term supplementation influences strength, endurance, balance, and exercise capacity in older adults and patients with heart failure.

Domoto et al (Domoto et al., 2024). conducted the earliest longitudinal assessment linking habitual taurine intake with age-related changes in physical fitness. Drawing on extensive data from the National Institute for Longevity Sciences – Longitudinal Study of Aging (NILS-LSA) cohort, more than 1,450 adults aged 40 years and older were followed over an eight-year period. Taurine intake was estimated through detailed dietary records linked to a large national food composition database, reflecting typical Japanese dietary patterns rich in seafood. The researchers monitored four indicators of physical performance: knee extension strength, sit-and-reach flexibility, one-leg standing time with eyes closed, and maximal walking speed. Their findings demonstrated a clear gradient across intake levels. Individuals in the highest tertile of taurine consumption maintained or slightly improved knee extensor strength, whereas those with the lowest intake exhibited measurable declines over the same period. These associations remained significant after adjusting for demographic variables, educational status, self-perceived health, depressive symptoms, and baseline fitness scores (Domoto et al., 2024). The protective effect of higher taurine intake was particularly pronounced among participants aged 65 years and older, suggesting that taurine may play an important role in mitigating age-related deterioration of lower-limb strength. By contrast, no meaningful associations were observed for flexibility, balance, or walking speed. Overall, this large-scale dataset indicates that routine dietary taurine contributes to the maintenance of quadriceps strength, an essential factor for preventing frailty, mobility impairments, and fall-related injuries in aging populations (Domoto et al., 2024).

In one of two complementary clinical investigations, Ahmadian et al (Ahmadian et al., 2017b). evaluated whether taurine supplementation could influence inflammatory and atherogenic biomarkers in patients with heart failure, both at rest and in response to graded exercise. Participants with reduced left ventricular ejection fraction (LVEF < 50%) were randomly assigned to receive either 500 mg of taurine three times daily or placebo for two weeks. Prior to and following the intervention, all subjects performed a multistage treadmill test adapted from the Bruce protocol, enabling simultaneous assessment of biochemical and functional responses to physical exertion. Supplementation resulted in notable reductions in markers of systemic inflammation, including CRP and platelet count, observed both before and after exercise (Ahmadian et al., 2017b).

Improvements were also seen in commonly used atherogenic indices castelli risk index I (CRI-I), castelli risk index II (CRI-II), and the atherogenic coefficient, which declined significantly only in the taurine group. Placebo-treated participants showed either no improvement or worsening of these measures over time. Although direct measures of exercise time or functional capacity were not primary outcomes, the more stable inflammatory and metabolic profile suggests that taurine creates a physiologically favorable environment for exertion in individuals with heart failure. Because inflammatory activation and vascular rigidity can limit exercise tolerance in this population, these results highlight taurine’s potential to support functional recovery by moderating systemic responses to physical stress (Ahmadian et al., 2017b).

In their second study, the same research group further examined taurine’s influence on cardiac electrical activity, myocardial oxygen use, and exercise performance (Ahmadian et al., 2017a). Sixteen patients completed a controlled, double-blind, two-week intervention with either taurine or placebo under an identical dosing schedule. This trial expanded beyond biochemical measurements to include electrocardiographic evaluations and direct assessments of exercise capacity. Following supplementation, several electrocardiogram (ECG) parameters shifted in a direction indicative of improved electrical stability. Specifically, Q-T intervals decreased and P-R intervals lengthened in the taurine group, suggesting more efficient atrioventricular conduction and enhanced electrophysiological resilience during exertion (Ahmadian et al., 2017a). In addition, taurine-treated patients demonstrated higher T-wave amplitudes and improved myocardial oxygen consumption following exercise, indicating better cardiac efficiency under stress. These changes were accompanied by significant gains in overall exercise tolerance, which were not mirrored in the placebo group. By improving electrical conduction, oxygen utilization, and functional output, taurine appears to enhance the cardiometabolic environment required for sustained physical activity in individuals with heart failure (Ahmadian et al., 2017a).

Further support for taurine’s ergogenic effects in compromised cardiac populations comes from the work of Beyranvand et al (Beyranvand et al., 2011), who investigated changes in exercise performance after a two-week period of supplementation. In their randomized, single-blind trial, patients with reduced LVEF underwent treadmill-based exercise tests both before and after treatment with taurine or placebo. Baseline characteristics, including exercise duration, metabolic equivalents (METs), and walking distance, were comparable between groups (Beyranvand et al., 2011). After supplementation, the taurine group displayed marked improvements across all functional outcomes, longer exercise time, higher MET levels, and greater total walking distance, while the placebo group showed no significant changes. These findings point toward taurine’s capacity to quickly enhance exercise performance, possibly through improved muscle contractile function, increased mitochondrial ATP availability, or more efficient hemodynamic responses. The magnitude and consistency of these improvements within only two weeks underscore taurine’s potential value in cardiac rehabilitation programs, where even small increments in physical capacity can meaningfully affect quality of life (Beyranvand et al., 2011).

Nasimi et al (Nasimi and Tadibi, 2025). examined the short-term, dose-dependent effects of taurine on physical performance in healthy older men. Although the study incorporated cognitive assessments, the focus here is solely on the physical outcomes. A single 6 g taurine dose led to significant improvements in dynamic balance (measured by Timed Up and Go test (TUG)), muscular endurance (chair-stand performance), and aerobic capacity (6-minute walk distance). No measurable benefit accompanied the 1 g dose, indicating a threshold effect for acute functional enhancements. These findings suggest that taurine may rapidly influence neuromuscular coordination and energy metabolism, with potential utility for older adults vulnerable to falls or mobility decline (Nasimi and Tadibi, 2025). Although taurine uptake into muscle and neural tissues is constrained by transporter availability, which typically adapts over longer time scales, the acute improvements reported by Nasimi et al. can be explained by mechanisms that do not rely on transporter−mediated intracellular accumulation. A high single oral dose produces a transient surge in circulating taurine, and this elevated plasma pool can exert rapid physiological actions, including modulation of calcium flux and excitation–contraction coupling, stabilization of cell membranes and osmotic balance, and short−term antioxidant or anti−inflammatory effects. These extracellular or near−membrane actions are capable of influencing neuromuscular excitability, coordination, and fatigue resistance within hours, providing a plausible rationale for the observed acute functional benefits independent of chronic transporter regulation or tissue loading kinetics.

Across a wide spectrum of study designs and populations including community-dwelling older adults and individuals with moderate to severe heart failure, taurine consistently demonstrates beneficial effects on physical performance. Long-term dietary intake supports the preservation of lower-limb strength, a critical determinant of mobility and independence. Short-term supplementation improves balance, endurance, and aerobic function in healthy older adults, while in heart failure patients, taurine enhances exercise tolerance, stabilizes cardiac electrophysiology, improves myocardial oxygen handling, and reduces inflammatory and atherogenic burden. Collectively, these findings indicate that taurine can influence multiple physiological systems that underlie physical functioning. Its combined effects on muscle performance, metabolic resilience, and cardiovascular stability position taurine as a promising complement to exercise-based approaches for maintaining functional capacity with aging.

### 3.4 Taurine and metabolic regulation: effects on glycemic control, lipid profiles, and body composition in aging

As individuals grow older, metabolic function gradually becomes less efficient, marked by greater abdominal fat deposition, impaired glucose handling, alterations in blood lipid levels, and a decline in resting energy expenditure. Together, these shifts create a metabolic environment that substantially increases vulnerability to cardiometabolic disease (Van der Leeuw et al., 2013). These changes are tightly linked to mechanisms including chronic low-grade inflammation, progressive insulin resistance, mitochondrial dysfunction, and alterations in adipocyte structure that become more pronounced with menopause and the gradual loss of muscle mass (Ross et al., 2002). Within this context, taurine has gained attention as a nutritional factor capable of influencing lipid handling, bile acid metabolism, glucose regulation, and inflammatory balance (Chen et al., 2012). The present section integrates evidence from clinical trials examining how taurine intake, alone or in conjunction with structured exercise programs, contributes to improvements in body composition, glycemic indices, and lipid regulation across populations such as postmenopausal women, women with type 2 diabetes, and older adults with sarcopenic obesity.

Buonani et al (Buonani et al., 2019). investigated the metabolic impact of taurine alongside a concurrent training model in postmenopausal women. Participants were randomly assigned to taurine alone, control, concurrent training plus taurine, or concurrent training plus placebo. Supplementation consisted of 1.5 g/day of taurine, and the exercise protocol combined resistance and aerobic modalities three times per week over eight weeks. Women who received taurine in combination with exercise showed the most pronounced improvements. This group demonstrated clear reductions in total and trunk fat and attained the greatest gains in lean body mass relative to both the control and taurine-only groups. Importantly, improvements in lipid markers, including meaningful reductions in total cholesterol and Low-Density Lipoprotein Cholesterol (LDL-c), occurred only when taurine accompanied the training sessions (Buonani et al., 2019). Although exercise alone improved some aspects of body composition, its effects were noticeably smaller without taurine. The minimal metabolic changes observed in the taurine-only group further emphasize that taurine’s influence is most evident when combined with structured physical training. Overall, these findings highlight a synergistic interaction in which taurine appears to amplify the cardiometabolic adaptations elicited by concurrent exercise in postmenopausal women (Buonani et al., 2019).

Building on this concept, Samadpour et al (Samadpour Masouleh et al., 2021). assessed how taurine supplementation integrates with TRX-based suspension training to modulate glycemic and lipid profiles in women with type 2 diabetes. Participants were randomized into four groups: TRX with taurine, TRX with placebo, taurine alone, and control. Over the eight-week intervention, supplemented groups consumed taurine three times daily, and a comprehensive panel of metabolic outcomes including fasting glucose, insulin, HbA1c, HOMA-IR, body composition indices, and lipid variables was evaluated (Samadpour Masouleh et al., 2021). Across all intervention groups, body mass, Body Mass Index (BMI), and body fat percentage declined; however, the largest reduction in body fat was observed in the TRX-plus-taurine group. Improvements in glucose regulation followed a similar pattern: while fasting glucose decreased significantly in both TRX conditions, the combined taurine-and-TRX intervention produced the greatest improvements in insulin levels and insulin resistance. Lipid outcomes reinforced this trend. Only participants receiving both taurine and TRX experienced substantial increases in high-density lipoprotein (HDL) alongside clear reductions in triglycerides and total cholesterol. Taurine alone produced modest metabolic benefits but lacked meaningful effects on glucose homeostasis without exercise. Collectively, these findings underscore taurine’s capacity to enhance the metabolic consequences of resistance-based exercise in women with type 2 diabetes (Samadpour Masouleh et al., 2021).

To further explore adipose-tissue–specific responses, Ortiz et al (Ortiz et al., 2025). examined how taurine paired with multicomponent training affects adipocyte morphology in older women with sarcopenic obesity, a condition characterized by a combination of excess body fat, diminished lean mass, and impaired metabolic flexibility. Participants underwent a 16-week intervention consisting of resistance, aerobic, and proprioceptive training three times weekly, accompanied by either 3 g/day taurine or placebo. Key outcomes included resting energy expenditure, circulating irisin, and direct measurements of adipocyte size using biopsies (Ortiz et al., 2025). The combination of taurine and exercise significantly reduced adipocyte area, indicating healthier fat-cell morphology and potentially improved metabolic function. Smaller adipocytes are less prone to inflammatory signaling and more responsive to metabolic stimuli, suggesting that taurine may facilitate adipose tissue remodeling when partnered with regular physical activity. Both exercise groups displayed increases in resting energy expenditure, with the taurine-supplemented cohort exhibiting the greatest rise. Although irisin levels showed an upward trend in the exercise groups, changes did not reach statistical significance. Even so, the overall pattern suggests that taurine can reinforce the cellular and metabolic adaptations triggered by multicomponent training in women with sarcopenic obesity (Ortiz et al., 2025).

Extending this work, Abud et al (Abud et al., 2025). focused specifically on glycemic responses and untargeted metabolomic changes following taurine supplementation with or without exercise in older women with sarcopenic obesity. Their 16-week intervention paralleled the Ortiz study, combining multicomponent training with daily taurine intake. Assessments included fasting glucose, insulin, homeostatic model assessment of insulin resistance (HOMA-IR), and advanced metabolomic profiling using Molecular Networking platforms (Abud et al., 2025). Exercise emerged as the predominant driver of improvements in glucose metabolism. The placebo-plus-exercise group demonstrated significant reductions in fasting glucose and insulin, while the taurine-plus-exercise group achieved similarly meaningful reductions in insulin and enhanced insulin sensitivity. Taurine without exercise did not improve glycemic parameters. Metabolomic analysis revealed decreases in circulating monosaccharides mainly in the exercise groups, indicating greater glucose utilization. Although taurine provided modest metabolic support, physical activity remained the central factor shaping glycemic outcomes. These results reinforce the idea that taurine may fine-tune metabolic adaptations but cannot replace the primary metabolic stimulus provided by exercise (Abud et al., 2025).

Taken together, the evidence demonstrates that taurine plays a supportive yet meaningful role in metabolic regulation across diverse aging populations, especially when combined with structured exercise. Consistent themes include reductions in adiposity, improved lipid markers, enhanced insulin sensitivity, and healthier adipocyte characteristics. While taurine alone yields limited metabolic benefits, its combination with physical training produces a more robust and multidimensional response. These synergistic effects highlight taurine’s potential as an adjunct nutritional strategy for optimizing metabolic health and body composition in older adults.

## 4 Glutamine supplementation and exercise in aging: integrative evidence from human trials

Glutamine is the most abundant free amino acid in circulation and contributes essential support to metabolic balance, immune activity, and muscle protein maintenance (Lacey and Wilmore, 1990). With aging, diminishing muscle mass, mitochondrial inefficiency, and hormonal alterations reduce endogenous production, leading to lower plasma levels. These declines can impair immune function, slow recovery after physiological stress, and worsen muscle integrity. Because older adults often exhibit weak anabolic responses and heightened inflammatory susceptibility, maintaining glutamine-related pathways has become an important research focus (Minet-Quinard et al., 1999; Breen and Phillips, 2011).

Exercise is a cornerstone strategy for healthy aging, yet it can be challenging for older adults due to reduced tolerance and slower recovery (Fell and Williams, 2008). Glutamine supplementation is therefore investigated as a potential aid to improve training adaptations, protect lean mass, and enhance metabolic and immune resilience (Candow et al., 2001). Glutamine also serves as a critical precursor for glutathione synthesis, the body’s principal intracellular antioxidant system. Age-related reductions in muscle mass and increased inflammatory signaling may limit glutathione production, increasing susceptibility to oxidative stress during metabolic challenges such as illness or exercise (Cruzat et al., 2018). A decline in endogenous glutamine production can therefore weaken immune responses, slow tissue repair, and increase vulnerability to oxidative injury. Because immune activation, ammonia detoxification, and muscle regeneration all intensify glutamine utilization, older adults—who already synthesize less glutamine, may encounter a mismatch between supply and metabolic need during illness, recovery, or repeated bouts of exercise (Marino and Calder, 2017). For this reason, glutamine is often regarded as “conditionally essential” during periods of high physiological stress (Lacey and Wilmore, 1990).

The following sections review randomized and controlled trials investigating glutamine combined with exercise, across three thematic categories. A consolidated summary of all extracted outcomes, population characteristics, and mechanistic insights is presented in **Table 2** to facilitate comparison across studies.

**Table 2:** Summary of randomized and controlled trials assessing glutamine’s physiological effects in elderly populations.

Study design & population characteristics	Sample size & glutamine intervention	Main findings	Mechanistic notes	Reference
RCT in elderly adults (~72–73 y); CET vs. non-practitioners; salivary redox and inflammation.	n=83; L-glutamine 0.3 g/kg/day for 30 days.	↑Uric acid (both Gln groups); ↓NO• in CET-Gln; CET better baseline IL-10/TNF-α; NP-Gln ↓GSSG; CET-Gln ↓GSH.	Strengthened oral redox balance; coordinated antioxidant shifts; CET enhanced response.	(Almeida et al., 2020)
RCT in elderly women (60–80 y) comparing exercising vs. sedentary participants.	n=44; glutamine 0.3 g/kg/day for 30 days.	↑GSH/GSSG; ↓fructosamine; ↑knee torque & power; ↓TBARs in all groups.	Improved glutathione turnover; enhanced glycemic and redox control.	(Amirato et al., 2021)
RCT in elderly (~72.6 y) receiving influenza vaccine; CET vs. non-practitioners.	n=84; 0.3 g/kg/day glutamine + maltodextrin × 30 days.	↑IgM/IgA in CET-Gln; ↑HI titers (highest in NP-Gln); ↑naive & effector CD4+ cells.	Enhanced humoral and cellular vaccine responses; stronger CD4 activation.	(Monteiro et al., 2020)
RCT in older CET practitioners vs. non-practitioners; HDL antioxidant enzyme function.	n=83; glutamine 0.3 g/kg/day for 30 days.	↑HDL-GPx & peroxidase (both Gln groups); ↑PON-1 only in CET-Gln; ↓oxidative ratios.	Reinforced HDL antioxidant defense; exercise primed PON-1 response.	(Pires et al., 2021)
RCT in elderly obese vs. adequate-weight adults; CET vs. non-practitioners.	n=84; glutamine 0.3 g/kg/day × 30 days.	CET-Gln ↑IL-10, ↓IL-6/IL-10; obese CET-Gln normalized IL-6; no glycemic/lipid effects.	Shift toward anti-inflammatory cytokine balance; lowered chronic inflammation.	(Sperandio et al., 2021)
RCT in TKA patients (~70.5 y) assessing recovery of quadriceps strength.	n=23; HMB+Arg+Gln for 5 days pre-op + 28 days post-op.	Maintained knee extensor strength; controls showed early strength loss; identical rehab.	Anti-catabolic postoperative support; preserved functional recovery.	(Nishizaki et al., 2015)
Double-blind RCT in elderly untrained men with T2DM; DOMS protocol.	n=40; glutamine 0.1 g/kg/day × 4 weeks.	↓CK at 72h (diabetic-Gln); earlier pain reduction; no effect on skin temperature.	Faster resolution of muscle membrane stress; reduced late-phase soreness.	(Biniaz et al., 2018)
Open-label RCT in adults ≥65 y undergoing cardiac surgery.	n=44; HMB 1200 mg + Gln 7 g + Arg 7 g for ≥2 weeks pre-op.	↑6MWD pre/post-op; ↑performance; ↓hospital stay; unchanged muscle mass.	Enhanced functional reserve; improved surgical resilience.	(Ogawa et al., 2025)
6−month RCT in healthy older adults (65–89 y).	n=31; daily HMB+Arg+Gln (3 g HMB, 14 g Arg, 14 g Gln).	↑Lean mass (DXA, ADP, 4−C model); ↑arm lean mass; improved stair climb; no ↑quadriceps volume.	Systemic anabolic support; neuromuscular efficiency improvement.	(Ellis et al., 2019)

RCT, randomized controlled trial; CET, combined-exercise training; TNF-α, tumor necrosis factor-alpha; NP-Gln, non-practitioner group receiving glutamine;GSSG, oxidized glutathione; IgM/IgA, immunoglobulin M/immunoglobulin A; CD4, cluster of differentiation 4 T lymphocytes; HDL-GPx, glutathione peroxidase activity in high-density lipoprotein; PON-1, paraoxonase-1; IL-6/IL-10, interleukin-6 to interleukin-10 ratio;HMB, beta-hydroxy-beta-methylbutyrate; Arg, arginine; Gln, glutamine; T2DM, type 2 diabetes mellitus; DOMS, delayed-onset muscle soreness; CK, creatine kinase; DXA, dual-energy X-ray absorptiometry; ADP, air displacement plethysmography. ↓ Decrease, ↑ Increase.

### 4.1 Pharmacokinetics

Glutamine exhibits a distinct pharmacokinetic profile characterized by rapid turnover, extensive first-pass utilization, and tight regulation between intestinal, muscular, immune, and renal compartments. As the most abundant free amino acid in plasma and muscle, it undergoes rapid turnover and extensive first-pass utilization by enterocytes and immune cells (Newsholme et al., 2003). Glutamine is rapidly absorbed in the small intestine through sodium-dependent and facilitative amino acid transporters, primarily members of the sodium-coupled neutral amino acid transporter (SNAT) and L-type amino acid transporter (LAT) families.

Following oral ingestion, plasma concentrations typically rise within 30–60 minutes and return toward baseline within several hours due to the high metabolic demand of enterocytes and immune cells. Because of its rapid cellular utilization, circulating glutamine displays a relatively short effective half-life and tightly regulated plasma concentrations, reflecting continuous exchange between tissues rather than long-term accumulation.

On the other hand, because a substantial portion of ingested glutamine is utilized within the intestinal mucosa during first-pass metabolism, the fraction reaching systemic circulation is relatively limited compared with many other amino acids. Intestinal uptake is driven by multiple transporter systems, including SNAT and LAT families, enabling high-capacity transfer but also significant presystemic metabolism (Rubio-Aliaga and Wagner, 2016). A large proportion of orally ingested glutamine is consumed by the gut and immune system before reaching circulation, which reduces its bioavailability relative to other amino acids (Cruzat et al., 2018). Skeletal muscle represents the largest endogenous reservoir of glutamine and functions as a central regulator of systemic glutamine availability. Other metabolically active tissues, including the liver, kidneys, immune cells, and intestinal epithelium, continuously exchange glutamine with the circulation, reflecting its role as a key nitrogen donor and metabolic substrate (Meynial-Denis, 2016).

This reliance on gut metabolism becomes particularly important in older adults, whose intestinal and immune demands are heightened due to chronic low-grade inflammation and altered barrier function. Age-related declines in muscle mass further reduce whole-body storage capacity, predisposing older individuals to fluctuations in plasma levels during metabolic stress (Wernerman, 2008).

With advancing age, several factors may alter glutamine kinetics, including reduced muscle mass, impaired protein turnover, and increased inflammatory signaling. These changes may shift glutamine balance toward increased utilization by immune and intestinal tissues while limiting the capacity of skeletal muscle to replenish circulating pools during periods of metabolic stress (Meynial-Denis, 2016). Renal handling also changes with aging, as reduced clearance and lower gluconeogenic activity shift glutamine nitrogen disposal patterns, influencing acid–base regulation and immune fuel availability (Holecek, 2013).

Exercise interacts with these pharmacokinetic features in meaningful ways. During prolonged or high-intensity exercise, skeletal muscle can release substantial amounts of glutamine into the circulation, supporting immune cell function and gluconeogenic pathways. However, repeated or strenuous exercise bouts may transiently reduce plasma glutamine concentrations, particularly in individuals with limited muscle reserves, which has been proposed as one factor contributing to exercise-associated immune perturbations (Castell and Newsholme, 1998).

### 4.2 Glutamine–exercise interactions in redox regulation and inflammatory control during aging

Progressive declines in antioxidant capacity and a chronic, smoldering inflammatory state are major contributors to functional impairments that emerge with advancing years (Martin and Grotewiel, 2006). Although regular exercise can attenuate many of these disturbances, its benefits may be limited when oxidative equilibrium is disrupted or when pro-inflammatory cytokines remain elevated (Pedersen et al., 2000).

Glutamine, a conditionally essential amino acid integral to glutathione synthesis, immune regulation, and cellular redox dynamics, has therefore gained attention as a potential nutritional support to enhance exercise-driven adaptations in older adults. In this context, recent clinical investigations provide insight into how glutamine behaves under exercise-related physiological demands.

Almeida et al (Almeida et al., 2020). examined whether L-glutamine could strengthen the redox and inflammatory benefits typically observed in older adults engaged in moderate combined (aerobic–resistance) training. The investigators compared non-practitioners with regularly exercising individuals during a 30-day supplementation period. Even before supplementation, the exercise group demonstrated a more favorable salivary inflammatory pattern, including reduced nitric oxide and TNF-α and increased IL-10 and uric acid in the serum. After glutamine intake, both active and sedentary participants showed elevated uric acid, whereas only the trained supplemented group displayed a reduction in nitric oxide, suggesting improved nitrosative regulation (Almeida et al., 2020). Alterations in glutathione status varied by training background: exercisers exhibited declines in GSH, while non-exercisers showed reductions in GSSG. Although salivary peroxidase activity declined in sedentary individuals, strong correlations between peroxidase activity and both glutathione and uric acid remained evident among exercisers, indicating a more coordinated antioxidant response. Indices such as trolox equivalent antioxidant capacity (TEAC), albumin, and reducing power were unchanged. Overall, the findings suggest that habitual training creates a more robust redox–inflammatory environment, and short-term glutamine supplementation can further support these favorable adaptations in older adults (Almeida et al., 2020).

Amirato et al (Amirato et al., 2021). investigated the influence of glutamine on oxidative balance, glucose regulation, and knee muscle performance in active versus sedentary elderly women. Prior to supplementation, non-exercising participants showed elevated fructosamine, insulin, and iron, along with lower strength and power, signaling impaired metabolic and neuromuscular status. After the 30-day intervention, both active and inactive women supplemented with glutamine exhibited clear reduction in GSSG, indicating enhanced glutathione turnover and improved antioxidant protection. A mild rise in uric acid was observed in active supplemented individuals, whereas iron concentrations were consistently lower in all physically active groups, underscoring the independent effect of exercise. Lipid peroxidation decreased in all groups, demonstrating broad improvement in oxidative control. Notably, combining glutamine with exercise produced the most pronounced gains in torque and power, while reductions in fructosamine and insulin were most evident in non-exercisers receiving glutamine. These results emphasize that glutamine improves antioxidant and glycemic parameters and, when paired with exercise, confers additional neuromuscular benefits in aging populations (Amirato et al., 2021).

Pires et al (Pires et al., 2021). focused on the interaction between glutamine supplementation and antioxidant enzyme function within HDL particles specifically glutathione peroxidase (GPx) and paraoxonase-1 (PON-1). Older adults who were either physically active or sedentary received glutamine or placebo for 30 days. Regardless of physical activity, glutamine significantly enhanced HDL-associated GPx and total peroxidase activity, accompanied by reductions in the ratios of peroxides to antioxidant enzymes and in iron-related oxidative indices. Interestingly, PON-1 activity increased exclusively in physically active participants receiving glutamine, suggesting that exercise primes HDL to respond more effectively to supplementation.

Glucose levels were consistently lower in active individuals, while plasma lipids, TEAC, uric acid, and TBARs were unaffected. These findings highlight glutamine’s capacity to reinforce antioxidant enzyme activity within HDL and demonstrate that exercise further augments PON-1 responsiveness, a key mechanism for vascular protection in older adults (Pires et al., 2021). Sperandio et al (Sperandio et al., 2021). investigated how glutamine interacts with inflammatory signaling in normal-weight and obese elderly subjects, stratified by exercise habits. Before supplementation, sedentary individuals exhibited higher BMI and elevated IL-6 in serum compared with exercisers. Following glutamine intake, the supplemented exercise group demonstrated a marked shift toward an anti-inflammatory profile, indicated by increases in IL-10 and a decreased IL-6/IL-10 ratio. When analyses were stratified by body weight, obese subjects consistently showed higher IL-6 levels at baseline; however, after supplementation, obese participants who exercised had IL-6 values similar to their normal-weight peers, suggesting that glutamine may help normalize inflammation in metabolically vulnerable groups. No significant changes were observed in lipid or glycemic indices, but the cytokine modulation indicates meaningful immunometabolic improvements. These outcomes suggest that glutamine can enhance exercise-related reductions in chronic inflammation, particularly in obese older adults who often display resistance to anti-inflammatory responses (Sperandio et al., 2021).

Across these clinical trials, a coherent pattern emerges: glutamine supplementation provides measurable benefits for redox regulation and inflammatory balance in aging populations, with the most pronounced effects occurring when combined with structured exercise. Glutamine contributes to improved glutathione metabolism, reduced lipid peroxidation, enhanced antioxidant enzyme activity in HDL, and a shift toward a more anti-inflammatory cytokine profile. Exercise independently strengthens these pathways, but the addition of glutamine appears to promote more synchronized antioxidant and anti-inflammatory responses and, in some cases, restores metabolic and inflammatory parameters in individuals with higher physiological risk. Improvements in muscle strength and power are also greatest when supplementation accompanies routine physical activity.

Collectively, these findings support glutamine as a promising nutritional adjunct to bolster exercise-induced enhancements in oxidative stability and inflammatory control during aging.

### 4.3 Glutamine-based nutritional strategies to enhance muscle strength, physical function, and recovery in older adults

The progressive loss of muscle capacity, slower post-exercise recovery, and heightened vulnerability to exercise-induced damage are common challenges in older adults (Li et al., 2024). Age-associated metabolic inflexibility, impaired protein turnover, and chronic comorbidities further diminish their ability to tolerate training stimuli (Shoemaker et al., 2022).

In this context, glutamine, alone or within multi–amino acid formulations, has been investigated as a supportive strategy for enhancing neuromuscular recovery, moderating soreness, and preserving overall physical function. The following clinical studies collectively illustrate how glutamine-based supplementation may influence muscle repair, functional performance, and postoperative recovery in aging individuals.

The investigation by Biniaz et al (Biniaz et al., 2018). assessed whether glutamine could modify the course of delayed-onset muscle soreness (DOMS) and biochemical markers of muscle damage in untrained older men with type 2 diabetes. Over four weeks, participants ingested either glutamine or placebo before performing an elbow-flexor resistance protocol specifically designed to trigger muscle microtrauma. Although serum CK increased in all groups, diabetic individuals receiving placebo showed the most pronounced and persistent elevations. In contrast, those supplemented with glutamine demonstrated a more rapid decline in CK values by 72 hours, suggesting improved clearance of muscle damage byproducts. Skin temperature, used as a proxy for inflammatory activity, rose similarly across all groups and was not influenced by supplementation. Pain intensity increased in most participants, yet diabetic men taking glutamine reported an earlier reduction in discomfort. Thus, while glutamine did not prevent DOMS, it appeared to shorten its duration and accelerate late-phase recovery, which may be clinically relevant for diabetic older adults who often experience extended recovery periods (Biniaz et al., 2018).

Building on the concept of post-exercise recovery, Nishizaki et al (Nishizaki et al., 2015). investigated whether a combined supplement containing β-Hydroxy-β-Methylbutyrate (HMB), arginine, and glutamine could help preserve quadriceps strength following total knee arthroplasty. Older adults consumed the supplement for several days before surgery and continued for nearly a month afterward while adhering to the same rehabilitation program. The control group experienced the typical sharp decline in knee extensor strength observed in the early postoperative phase. In contrast, the supplemented group-maintained muscle strength much closer to preoperative values and showed a smoother recovery trajectory over the subsequent weeks. Importantly, neither total energy expenditure nor non-operated limb strength differed between groups, indicating that the beneficial effects cannot be attributed to differences in physical activity. These findings suggest that targeted amino acid support may help reduce postoperative muscle weakness, one of the primary barriers to early mobility and functional independence following orthopedic surgery (Nishizaki et al., 2015).

Extending the lens to major cardiac procedures, Ogawa et al (Ogawa et al., 2025). evaluated the impact of preoperative HMB/arginine/glutamine supplementation on recovery after cardiac surgery in older patients. Supplementation for at least two weeks before surgery resulted in substantially higher six-minute walk distances both preoperatively and during early postoperative assessment, reflecting superior cardiopulmonary reserve and improved resilience to surgical stress. Indicators of muscle strength and overall functional performance also improved meaningfully in the supplemented group, despite no observable differences in muscle mass. Notably, the intervention group experienced a shorter hospital stay, a clinically meaningful outcome in cardiac surgery patients who are at high risk for postoperative complications. No adverse effects were reported, reinforcing the safety of this multi–amino acid strategy in medically fragile, older adults. Taken together, these results highlight the potential for targeted nutritional support to enhance postoperative functional outcomes without requiring changes in body composition (Ogawa et al., 2025).

Further supporting these observations, Ellis et al (Ellis et al., 2019). conducted a six-month trial examining long-term supplementation with a mixture of HMB, arginine, and glutamine in healthy older adults. Participants receiving the supplement demonstrated significant gains in total lean body mass across several assessment techniques, including dual-energy X-ray absorptiometry (DXA) and a four-compartment model. Improvements also emerged in functional performance, particularly in the timed stair climb—a task closely aligned with everyday mobility. Interestingly, quadriceps muscle volume assessed via magnetic resonance imaging (MRI) remained unchanged, suggesting that functional improvements may stem from neuromuscular adaptations or distributed increases in lean mass rather than localized hypertrophy. Additional increases in arm lean mass reinforce this broader anabolic effect. Overall, the study indicates that multi–amino acid supplementation may help counteract gradual declines in muscle mass and functional capacity in otherwise healthy older adults (Ellis et al., 2019).

Considering the collective evidence from these clinical trials, glutamine either by itself or within formulations that include HMB and arginine demonstrates consistent benefits for muscle recovery and functional performance in older populations. Supplementation reduced the duration of DOMS in diabetic individuals, attenuated postoperative strength loss after orthopedic surgery, improved walking capacity following cardiac procedures, and supported increases in lean mass among community-dwelling elders. Although individual mechanisms differ, shared themes include improved protein turnover, reduced muscle membrane stress, and enhanced metabolic resilience in the face of physical stressors. Notably, the greatest functional advantages emerged when supplementation was paired with structured rehabilitation or habitual activity, underscoring the role of nutrient support as a complement to physical training in promoting independence and mobility in aging adults.

Studies employing multi-ingredient supplements should be interpreted with caution when attempting to determine the specific contribution of taurine or glutamine. Because these formulations contain several bioactive compounds that may independently influence metabolic, inflammatory, neuromuscular, or cognitive outcomes, it becomes difficult to attribute observed effects to any single component. Potential synergistic or additive interactions among ingredients may further confound interpretation, making it unclear whether the reported benefits are driven by taurine or glutamine, by other constituents of the formulation, or by their combined effects. Consequently, while such studies may provide useful preliminary insights into the potential benefits of complex nutritional interventions, they offer limited mechanistic clarity regarding the independent roles of taurine or glutamine. For this reason, findings derived from multi blend supplements should be considered less definitive than studies examining these amino acids in isolation or in clearly controlled combinations with exercise.

## 5 Practical applications and evidence-informed recommendations for taurine and glutamine supplementation in aging

The evolving evidence on taurine and glutamine supplementation in conjunction with exercise suggests that these amino acids may serve as useful adjuncts for improving physiological resilience in older adults. Although study designs remain heterogeneous, several practical insights can be distilled for real-world application, particularly regarding dosing strategies, target populations, and integration with structured physical activity.

Taurine has been examined across a wide dose spectrum, from habitual dietary intake to acute supplemental ingestion of 1–6 g. Human studies consistently report that doses up to 6 g are well tolerated, with no adverse effects noted even in older adults. Notably, a single 6 g dose improved balance, muscular endurance, aerobic capacity, and cognitive performance in men aged 60–69, whereas 1 g failed to elicit measurable effects (Nasimi and Tadibi, 2025). A meta-analysis further confirmed that taurine enhances endurance performance across doses of 1–6 g, with no clear difference between acute and short-term supplementation protocols (Waldron, 2018). Chronic intake at 1.5–3 g/day has also been used safely in postmenopausal women, adults with type 2 diabetes, and older women with sarcopenic obesity (Buonani et al., 2019; Samadpour Masouleh et al., 2021; Ortiz et al., 2025). Together, these data indicate that taurine possesses a favorable short-term safety profile, although most evidence comes from relatively brief interventions.

Glutamine supplementation shows a similarly reassuring safety pattern, with typical doses ranging from 0.1 g/kg/day in single-compound trials to approximately 14 g/day in multi–amino acid blends. No study involving older adults, including diabetic men, postmenopausal women, or patients recovering from orthopedic or cardiac surgery, reported supplementation-related adverse events (Nishizaki et al., 2015; Biniaz et al., 2018; Ogawa et al., 2025). Still, because many trials excluded participants with advanced renal, hepatic, or cardiovascular disease, the safety of glutamine in frail or medically complex older adults requires cautious interpretation and ideally clinical supervision.

The populations that benefit most from taurine or glutamine supplementation share features of reduced metabolic flexibility, attenuated antioxidant capacity, chronic inflammation, or impaired functional reserve. Taurine appears particularly relevant for individuals with compromised aerobic capacity, balance deficits, metabolic dysregulation, or heart failure, where improvements in inflammatory markers, cardiac efficiency, and exercise tolerance have been repeatedly documented (Beyranvand et al., 2011; Ahmadian et al., 2017b). Enhanced lipid regulation, reduced trunk fat, and improved glycemic control have also been observed when taurine accompanies structured training in postmenopausal women and individuals with type 2 diabetes (Samadpour Masouleh et al., 2021).

Glutamine’s benefits are most consistent in subgroups with elevated inflammatory load, weakened antioxidant defenses, or reduced recovery capacity. Studies show that supplementation, especially when paired with exercise, strengthens glutathione metabolism, increases HDL-associated antioxidant enzyme activity, and improves redox balance (Almeida et al., 2020; Pires et al., 2021). In clinical contexts, multi–amino acid formulations containing glutamine, HMB, and arginine help preserve strength after orthopedic surgery, enhance walking capacity in cardiac patients, and contribute to favorable changes in whole-body lean mass among healthy older adults (Nishizaki et al., 2015; Ellis et al., 2019). Moreover, combining glutamine with habitual exercise enhances immune responsiveness to influenza vaccination, particularly in sedentary older adults (Monteiro et al., 2020). Across nearly all trials, the most consistent pattern is that taurine and glutamine exert synergistic rather than standalone effects. Supplementation tends to potentiate exercise-induced improvements rather than substitute for physical training.

Because exercise remains the primary determinant of functional and metabolic adaptation in aging, supplementation should be integrated into, rather than replace, structured training. In practice, a conservative and individualized approach is advisable. Short-term use of 1.5–3 g/day taurine appears safe and potentially beneficial for older adults participating in regular exercise, while acute 6 g dosing may be appropriate for targeted performance or cognitive–motor enhancement in healthy individuals.

Long-term use at higher doses should be approached cautiously due to limited data. Glutamine supplementation may be considered for older adults engaged in exercise or rehabilitation programs, though individuals with chronic renal or hepatic impairment should receive medical evaluation before use. Finally, supplementation should be embedded within broader lifestyle strategies that include regular physical activity, balanced nutrition, adequate protein intake, and management of comorbid conditions. When used judiciously, taurine and glutamine may help refine exercise adaptations, support recovery, and strengthen physiological systems that decline with advancing age; however, they should be viewed as complementary tools within an integrated approach to healthy aging rather than universal solutions.

## 6 Limitations and future directions

Despite promising findings on taurine- and glutamine-based supplementation in older adults, the existing literature is constrained by several methodological and mechanistic limitations that restrict the strength and generalizability of current conclusions. Many trials have small sample sizes, often fewer than 20 participants per treatment arm, reducing statistical power and increasing the likelihood that observed benefits may reflect variability rather than true physiological adaptation. Short intervention periods, frequently between 2 and 16 weeks, also limit the ability to assess sustained effects on cognition, muscle performance, metabolic regulation, or immune function. These short time frames are particularly limiting for processes such as sarcopenia progression and neuroimmune remodeling, which typically require longer observation windows.

Considerable heterogeneity across studies further complicates interpretation. Exercise modalities range widely across investigations, including concurrent training in postmenopausal women (Buonani et al., 2019), TRX suspension training in individuals with type 2 diabetes (Samadpour Masouleh et al., 2021), and multicomponent protocols in sarcopenic obesity (Abud et al., 2025; Ortiz et al., 2025). This variability makes it difficult to determine whether taurine and glutamine exert exercise-specific effects or whether their benefits generalize across training types. Supplementation protocols also differ substantially.

Taurine doses vary from chronic intake of 1.5–3 g/day to acute single boluses of 6 g (Nasimi and Tadibi, 2025), while glutamine trials use doses from 0.1 g/kg/day to multi-nutrient blends containing HMB and arginine (Nishizaki et al., 2015; Ellis et al., 2019). Such inconsistencies prevent clear identification of optimal doses, timing strategies, or whether improvements are driven by the amino acid itself or by interactions with other ingredients.

Limitations in biomarker selection and mechanistic assessment also narrow the interpretive scope of current research. Many trials rely on basic oxidative stress or inflammatory markers, yet use different assay methods, sampling time points, or units of analysis, complicating direct comparison.

Cognitive outcomes are often restricted to brief screening tools such as the MMSE, with little incorporation of mechanistic indicators like neurotrophic factors, neuroinflammatory profiling, or neuroimaging data. In taurine studies, proposed mechanisms related to cortical plasticity and inhibitory interneuron activation remain largely derived from animal research rather than human trials (Gawryluk et al., 2024).

Similar concerns apply to glutamine, where biochemical improvements in glutathione turnover or HDL-associated antioxidant enzymes (Pires et al., 2021) are not consistently paired with clinical measures such as mobility, hospitalization outcomes, fall risk, or quality of life. Population-level constraints also limit generalizability. Many studies exclude individuals with multimorbidity, advanced renal or hepatic dysfunction, recent surgeries (except in targeted trials such as (Nishizaki et al., 2015; Ogawa et al., 2025)), cancer history, or significant cardiovascular disease.

As a result, current evidence may underestimate potential risks or overestimate benefits in populations with high clinical complexity. Sex differences remain insufficiently explored despite indications that inflammatory and metabolic responses to both exercise and supplementation may diverge between men and women. These limitations underscore the need for more rigorous research. Future work would benefit from larger, longer-duration randomized controlled trials capable of assessing clinically meaningful endpoints. Standardization of dosing strategies is important, particularly for distinguishing chronic lower-dose taurine (1.5–3 g/day) from higher acute doses such as 6 g, as well as isolating the effects of glutamine from those of multi-ingredient blends. Harmonized exercise protocols would enable clearer evaluation of supplement–exercise interactions. Deeper mechanistic exploration is also needed.

Integrating metabolomics, lipidomics, redox profiling, and immune phenotyping could help identify biological signatures of response. Neuroimaging and cerebrovascular assessments are warranted to test hypotheses related to taurine’s effects on cognitive–motor coupling and BBB stability. For glutamine, more targeted trials are needed to clarify its independent roles in glycemic regulation, immune responsiveness to vaccination (Monteiro et al., 2020), postoperative recovery (Nishizaki et al., 2015; Ogawa et al., 2025), and oxidative balance in vulnerable metabolic phenotypes.

Future research should also include populations that have been underrepresented, frail older adults, individuals with multiple chronic diseases, and adults undergoing structured rehabilitation, while monitoring long-term safety, particularly for high-dose taurine and glutamine use in those with impaired metabolic clearance.

Collectively, advancing the field will require harmonized methodologies, multi-dimensional mechanistic analyses, and clinically relevant outcomes that reflect the complex physiological demands of aging. These enhancements will allow taurine and glutamine to be rigorously evaluated as components of integrated lifestyle and rehabilitation strategies aimed at supporting healthy aging.

## 7 General conclusion

Across the current body of evidence, taurine and glutamine emerge as two conditionally relevant amino acids with potential to modulate several domains of age-related physiological decline. Although the available trials are limited in number, heterogeneous in methodological design, and often involve modest sample sizes, consistent patterns across studies indicate that both compounds may serve as adjunctive, not primary, agents that enhance the benefits of structured exercise, rehabilitation programs, and other lifestyle-based strategies in older adults.

Taken together, the findings suggest that taurine and glutamine support overlapping yet distinct biological pathways involving redox balance, inflammatory regulation, neuromuscular performance, metabolic function, and immune responsiveness.

The present review is among the first to synthesize evidence from both amino acids in a unified framework, highlighting shared mechanisms and differential domains of effectiveness across aging physiology. Taurine appears particularly relevant in contexts linked with neuroimmune function, cardiopulmonary performance, and metabolic resilience. Across neuroimmune outcomes, taurine supplementation has been associated with reductions in inflammatory and proteolytic enzymes, partial stabilization of cognitive measures, and improvements in cognitive–motor integration. In elderly women, taurine lowered MPO and MMP-9 and helped preserve cognitive scores, either independently or alongside exercise (Chupel et al., 2021). Acute high-dose ingestion in older men enhanced balance, endurance, and cognitive–motor coupling, suggesting potential short-term neurometabolic benefits that may reflect improved mitochondrial and calcium-handling dynamics (Nasimi and Tadibi, 2025). Experimental work further strengthens the biological plausibility of these effects: taurine restored experience-dependent cortical plasticity in aged animals without directly altering glutamate/GABA ratios, instead enhancing activity of somatostatin-expressing interneurons essential for learning-related plasticity (Gawryluk et al., 2024).

Beyond neural pathways, taurine has shown promising associations with physical performance. Longitudinal evidence indicates that higher habitual taurine intake is linked with better preservation of knee extensor strength in adults over 65 years, suggesting a possible protective role in lower-limb musculoskeletal aging (Domoto et al., 2024). Clinical trials in heart failure populations provide additional insight, where short-term taurine supplementation improved inflammatory and atherogenic profiles, enhanced cardiac electrical stability, and increased treadmill exercise capacity (Beyranvand et al., 2011; Ahmadian et al., 2017a). Despite the encouraging nature of these results, their generalizability remains limited by small sample sizes and short intervention durations. In metabolic studies, taurine often acts synergistically with exercise.

In postmenopausal women, taurine amplified training-induced reductions in adiposity and improvements in lipid markers (Buonani et al., 2019). Women with type 2 diabetes experienced greater improvements in insulin regulation and lipid metabolism when taurine was paired with resistance-based suspension training (Samadpour Masouleh et al., 2021). Similarly, in older women with sarcopenic obesity, taurine enhanced exercise-induced remodeling of adipocyte morphology and increased resting energy expenditure (Ortiz et al., 2025), while metabolomic findings suggest that taurine may modulate glucose utilization without replacing exercise as the primary driver of metabolic change (Abud et al., 2025).

Additional research using multi-ingredient blends containing taurine indicates modest improvements in inflammatory mediators, strength, and perceived energy, underscoring its potential as part of broader integrative supplementation protocols (Dunn-Lewis et al., 2011).

Parallel to taurine, glutamine demonstrates a supportive role across oxidative, inflammatory, neuromuscular, and immune pathways in aging populations. Evidence indicates that glutamine supplementation enhances glutathione metabolism, reduces lipid peroxidation, and strengthens HDL-associated antioxidant enzymes, especially in physically active individuals (Almeida et al., 2020; Pires et al., 2021). In metabolically vulnerable groups such as obese older adults, glutamine further aided normalization of inflammatory markers and improved IL-6/IL-10 balance—effects that aligned most strongly with concurrent physical activity (Sperandio et al., 2021).

Glutamine also contributes meaningfully to neuromuscular recovery and functional maintenance. Although it does not prevent delayed-onset muscle soreness, supplementation accelerated the decline of serum CK levels and reduced late-phase discomfort in diabetic older men following eccentric exercise (Biniaz et al., 2018). Multi-amino acid formulations combining glutamine with HMB and arginine demonstrated clinically relevant benefits in surgical populations. Older adults undergoing total knee arthroplasty exhibited attenuated postoperative strength loss, while cardiac surgery patients receiving preoperative supplementation achieved greater walking distances and shorter hospital stays (Nishizaki et al., 2015; Ogawa et al., 2025). Longer-term use also increased whole-body lean mass and improved stair-climbing performance in healthy older adults, despite stable quadriceps volume, suggesting functional neuromuscular adaptations rather than localized hypertrophy (Ellis et al., 2019). Glutamine’s immunomodulatory effects add another dimension to its potential utility.

Supplementation alongside exercise enhanced both humoral and cellular responses to influenza vaccination, yielding greater antibody production, higher hemagglutination inhibition titers, and stronger expansion of naïve and effector CD4^+^ T-cell subsets, with particularly pronounced benefits in sedentary participants (Monteiro et al., 2020). These findings indicate that glutamine may be able to partially counteract immunosenescence in older adults, especially among those with lower baseline immune competence.

Collectively, the converging evidence positions taurine and glutamine as adjunctive nutritional strategies that may reinforce physiological domains commonly weakened with age. Although their mechanisms and primary spheres of influence differ, both compounds consistently demonstrate greater efficacy when combined with exercise, rehabilitation, or structured lifestyle interventions.

This synergistic pattern suggests that neither amino acid functions as an isolated therapeutic agent; instead, they may optimize or amplify the body’s adaptive responses to physical and metabolic stimuli. Importantly, the present synthesis highlights a novel conceptual framework by jointly evaluating taurine and glutamine across shared dimensions of aging biology.

Nevertheless, definitive recommendations remain premature, and future trials should address optimal dosing, long-term safety, population-specific responses, and combined supplementation strategies. Despite these limitations, current evidence supports the view that taurine and glutamine may meaningfully contribute to integrative, lifestyle-oriented approaches aimed at promoting healthier aging.
